# Burden associated with Fabry disease and its treatment in 12–15 year olds: results from a European survey

**DOI:** 10.1186/s13023-022-02417-3

**Published:** 2022-07-15

**Authors:** Lisa Bashorum, Gerard McCaughey, Owen Evans, Ashley C. Humphries, Richard Perry, Alasdair MacCulloch

**Affiliations:** 1 Amicus Therapeutics UK, One Globeside, Fieldhouse Lane, Marlow, SL7 1HZ UK; 2 Adelphi Values PROVE, Adelphi Mill, Grimshaw Lane, Macclesfield, SK10 5JB UK

**Keywords:** Fabry disease, Adolescent burden, Caregiver burden, Enzyme replacement therapy

## Abstract

**Background:**

Fabry Disease (FD) is a rare X-linked metabolic lysosomal disorder. FD has a broad range of symptoms which vary markedly between patients. The heterogenous nature of the disease makes diagnosis difficult for health care professionals (HCPs), which in turn has a significant effect on the patient’s quality of life (QoL). As few adolescent patients are eligible for treatment, to date there has been little published data on the burden of disease and impact of treatment on these patients and their caregivers. This study was developed to provide some insight into these groups.

**Methods:**

An online-based survey was performed to gather further insights on the burden of FD in 14 adolescents aged 12–15 years old across three European countries, from the perspective of the patients, caregivers and HCPs.

**Results:**

Symptom burden was found to be high in the adolescent population, with ‘pain’ and ‘intolerance to heat or cold’ commonly reported symptoms, both by patients and to HCPs. Eleven of the 14 patients surveyed were receiving enzyme replacement therapy (ERT), with their post-ERT symptomology showing improvement when compared to symptoms before receiving ERT. The majority of caregivers believe their child’s overall health has improved since starting ERT. While there was a positive outlook towards ERT noted by the patients and caregivers, 4/5 HCPs believed there is ‘a need for more efficacious treatment options’ and all HCPs noted that there is ‘a need for more manageable treatment options’. FD was shown to place a burden on caregivers, who reported feelings of guilt and absences from work.

**Conclusions:**

Data show there is a significant symptom burden for the adolescent, which affects their QoL and mental health, as well as placing a burden on the wider family. While ERT is an effective treatment and provides symptom relief for many of the respondents in the survey, they still reported symptom burden. Additionally, there was reporting of reluctance to engage in treatment or difficulties associated with the treatment. Heterogeneity in symptom presentation suggests that the treatment regimen needs to be tailored to the individual. Physicians therefore need to have a choice of treatment options available to help them manage symptoms and disease where the benefit to risk ratio is in favour of undergoing treatment.

**Supplementary Information:**

The online version contains supplementary material available at 10.1186/s13023-022-02417-3.

## Background

Fabry disease (FD) is rare X-linked metabolic lysosomal disorder, caused by pathogenic variants of the gene *GLA* [1]. Pathogenic variants of *GLA* are defective, or lead to complete loss of function of the enzyme α-galactosidase A (α-Gal A), which is involved in metabolic hydrolysis of the glycosphingolipid globotriaosylceramide (GL-3) [2]. As such, defects in α-Gal A lead to a lack, or complete loss, of GL-3 breakdown, causing its progressive accumulation, and consequent ER stress and oxidative damage, which disrupts cell function [2–5]. In addition to causing vessel occlusion, tissue ischaemia and fibrosis, it is believed that accumulation as well as the ischaemia itself promotes the release of secondary mediators [6, 7]. These numerous secondary mediators result in a variety of effects including chronic inflammation, which plays a key role in the pathogenesis of FD-related organ damage [6–10].

Disease progression is influenced by the biological sex of the individual, genotype and the presentation of symptoms, with a wide range in heterogeneity observed [7]. Newborn screening (NBS) studies have revealed ‘classic FD’ occurs at frequencies of up to 1 in 22,570–37,000 in males, it presents with a large spectrum of symptoms, and generally has early onset with low activity of α-Gal A [11–13]. ‘Later onset/atypical FD’ is usually limited to one organ system with partial α-Gal A activity (2–20% of normal) seen. In ‘later onset/atypical FD’, NBS studies have reported its frequency at up to 1 in 1-3100–1,390 males [8, 11, 13]. Disease severity varies in heterozygous females, ranging from asymptomatic to a severe form which resembles the male ‘classic’ phenotype. It has been estimated that the severe form is exhibited in around half of female FD patients, and two-thirds of males [14].

Accumulation of GL-3 begins in utero, and the degree of accumulation depends on the level of *α*-Gal A present in the individual [5]. The onset of FD symptoms typically occurs at an earlier age in males, with a median age of 6 as compared to 9 in females [15]. Key presenting symptoms in childhood have been reported as neuropathic pain and gastrointestinal (GI) symptoms [15]. GI problems have been reported as early as 1 year-old, and neuropathic pain among other symptoms have been observed from 2 years of age [16]. Other symptoms, such as renal and cardiac disease typically manifest later during adolescence or in adulthood [17]. Overall, the range of symptoms of FD are extensive and symptoms manifest differently in each individual making it difficult to diagnose with high levels of misdiagnosis seen [18]. The heterogeneous nature, as well as rarity of the disease means specialist intervention is usually required [18].

Diagnosis is typically delayed in childhood, especially in the absence of family history. Diagnosis is often not made until the disease has progressed for years, at which point organ damage has already occurred. For instance, the Fabry outcome survey reported the delay between onset and diagnosis was 12 years in both males and females [19, 20]. Evidence shows there is an increased psychological impact among patients whose clinical symptoms, such as fatigue and heat intolerance were either dismissed or misdiagnosed. Therefore, early diagnosis of symptomatic children prior to onset of irreversible damage is ideal, which may also help to prevent complications and enhance well-being [17, 21].

FD burden has been explored in adults, with description of humanistic burden due to the wide range of body systems that can be affected leading to multiple health problems that can directly impact the patient’s quality of life (QoL) [2, 8, 22, 23]. This is particularly pertinent considering patients are faced with a lifelong, incurable, debilitating and progressive disease, which has both psychological as well as physical consequences [24]. However, the specific physical and emotional burden caused by FD in adolescents has to date not been well characterised. This is due in part to the rarity of the disease and relatively recent recognition of its early disease manifestation and progression. There is a particular need for research to be done in this younger population, as early diagnosis and intervention could benefit adolescents significantly as they progress into adulthood [2].

While treatment options that have proven to be effective are available in adults, approved treatment options are relatively limited in the adolescent population. Oral chaperone therapy has now received a European license expansion in 12-15 year olds who weigh more than 45 kg [25]. The current standard of care for FD in adolescents is enzyme replacement therapy (ERT), with two products available as of 2022, one available in the EU and Japan, and the other approved for use in the US, Japan and the EU [26–29].

ERT provides a functional form of the *α*-Gal A enzyme, to allow normal metabolic processes to occur, leading to the breakdown of GL-3 [30]. ERT is a lifelong treatment that is preventative and cannot reverse damage that has already occurred. ERT is administered intravenously every two weeks, and can place a burden on the patient, caregiver, and healthcare provider [31]. The efficacy and safety of ERT in children has been investigated and demonstrated in studies. Treatment has been shown to reduce accumulation of GL-3, improve pain, GI symptoms, QoL, energy and activity levels [32–41].

ERT in some cases can be administered at home, however medical specialist support is still needed for intravenous administration, as well as the temperature-controlled transport of the delivery [42]. In both the clinic and home settings, the process of receiving ERT leads to absence from school, and in many cases, absence from work for the caregiver [43].

Although well tolerated overall, ERT therapy can be associated with adverse events such as infusion associated reactions (IARs) which can cause delay or discontinuation of treatment. Administration of ERT can also be associated with antibody formation that can interfere with ERT efficacy and exacerbate IARs [44]. In addition, it may not be suitable for some patients who are ‘needle phobic’, which is common in adolescents [45]. The decision to begin treatment rests on symptom control as well as the burden of the treatment itself e.g. the time commitment and potential side effects, and often requires a joint discussion between the family and all the key healthcare professionals (HCPs), in order to ensure the best interests of the adolescent are served. However, ERT can greatly improve the QoL for many patients, with few side effects, allowing the patient to lead a more fulfilling life with fewer challenges caused by the symptoms of FD.

The aim of our study was to capture the experience of adolescents with FD by obtaining data using online surveys. These surveys were designed to gather an understanding and appreciation of any burden of FD itself, as well the impact of treatment. Due to the wide impact of FD, both the perspective of the adolescent patient as well as their caregiver was sought with separate surveys for each. Further, due to the specialised nature of managing FD care, expert HCPs were also enlisted to answer questions for a third survey. The multi-perspective surveys allowed for the first time a comprehensive understanding of the burden of adolescent FD not only on the patient, but also their caregivers, as well as an appreciation of correlation and differences between patient, caregiver and HCP views. Overall, the study highlights where improvements could be made for this patient population.

## Methods

### Design overview

A non-interventional study was conducted from June 2021 to September 2021, to explore the potential burden of FD in adolescent patients in three European countries; France, Germany and the UK. Primary data were collected using online surveys targeted to patients with FD, their caregivers, and HCPs with experience in this therapeutic area and patient population.

Survey questions were designed to capture if there was burden from FD itself i.e. the level of symptomology experienced and impact on daily life and mental outlook, as well as the experience of undergoing treatment from each stakeholder’s perspective.

The survey for the adolescent patients with FD consisted of questions composing of agreement scores, multiple choice, and a limited number of free response answers to capture information regarding the respondent’s reasoning. The questions were designed to ascertain if there was burden from FD and its treatment from the perspective of the adolescent patient, and where this was identified to characterise it.

The caregiver survey consisted of questions capturing the potential psychological, financial, social and physical burden of caring for a child with FD. The survey also assessed the severity of the symptoms of FD in the child from the perspective of the caregiver. The format consisted of agreement scores, multiple choice and a limited number of free response answers.

The survey provided to the HCPs consisted of questions created to understand potential burden of FD on both the patient and caregiver, from their perspective. It also explored the current management of FD, including willingness to prescribe the standard of care and any unmet needs.

### Participant recruitment

A total of 14 patients, 14 caregivers and five HCPs from France, Germany and the UK were surveyed, with recruitment performed by a third-party recruiter, to ensure appropriate blinding. One respondent was screened out due to ineligibility, as they were outside the age range parameters. Patient advisory organisations (PAOs), in each market also provided recruitment support and assisted in identifying relevant individuals for the patient and caregiver surveys. Participant recruitment was achieved via email advertisement, to each PAOs’ active members. The following PAOs provided this support; The MPS Society—Society for Mucopolysaccharide Diseases (UK), APMF—Association des Patient de la Maladie de Fabry (France) and MFSH—Morbus Fabry Selbsthilfegruppe (Germany). As well as providing recruitment support, the PAOs reviewed and contributed to survey development to ensure comprehensive and relevant questioning that was appropriate for the age group. Online surveys were provided in the respondents’ native language. Where appropriate, respondents were offered remuneration at fair market value in the form of a wire transfer or Amazon gift vouchers as compensation for their time completing the surveys.

Respondents for the patient survey were recruited based on the following inclusion criteria; aged 12–15 years old, diagnosed with FD by a physician, and eligible for Fabry treatment. An associated caregiver of the patient was subsequently recruited to respond to the caregiver survey. Due to the specific inclusion criteria of our survey, the eligible respondents for the survey were extremely limited. Of note there are only around half a dozen specialised centres treating paediatric patients in Germany, and a fraction of these patients are receiving Fabry-specific therapy. Likewise, there are only four regional specialised centres that provide adolescent services for Fabry in the UK and 10 centres that follow adolescent Fabry patients in France, but not all of these provide treatment. As such there are only 10s of patients undergoing treatment in the 12–15 year old age group, and few specialists with knowledge of their care. Fifteen HCPs considered as specialists in treating adolescent patients with FD were invited to participate in the study. HCPs were invited to participate via a third-party recruiter, specific reasons for decline in participation were not noted.

### Data analysis

All analysis was performed using anonymised data. The frequency and proportion of respondents recording a response was captured for multiple choice questions. Quantifiable information from respondents was also captured using different answer scales. For reporting on symptom burden Likert scoring was selected, this enabled conversion of survey answers reporting on frequency to numeric values for analysis and comparison. The frequency of each agreement score from individual rating statements was compiled to observe the distribution of ratings across the respondent base. These scores were examined in relation to the overall distribution of scores, as well as to the participants who were in high agreement (scored 8–10), or low agreement (scored 1–3). Statistical analysis was not carried out on the dataset due to the small sample size.

## Results

### Sample composition

Surveys were completed by 14 adolescent patients with FD. Of the 14 patients, seven were from the UK, five from France and two from Germany. Of these 14 patients, two females were not receiving any specific treatment for their disease and of the 12 receiving treatment (eight male and four female), 11 were receiving ERT (eight male and three female). Five patients who were receiving ERT were first treated at 0–10 years old (4/5 were male), two males at 11 years old, two males at 13 years old and two females at 15 years old. In total, eight of the 14 patients were male and six were female. Further information related to age at diagnosis, time since last infusion and additional medication taken are reported in Additional file 1: Table S1.

An associated caregiver for each patient provided responses to the survey, and therefore 14 caregiver responses were received. Of the five French respondents, three caregivers were in the age group 40 and  < 50 years old and two were over 50 years old. The two German caregivers were in the age groups 30- < 40 years old and 40 and  < 50 years old. One caregiver from the UK was between 30 and  < 40 years old, and six were between 40 and  < 50 years old. Eleven of the 14 caregivers were female, with one male caregiver from France and two from the UK. Nine of the 14 caregivers also suffered with FD (eight female and one male) (Additional file 1: Table S1).

Survey responses were received by five HCPs, one from France, one from Germany and three from the UK. HCPs self-described their clinical role in regard to FD as either the clinical lead or noted involvement in diagnosis, monitoring, and treatment.

### Patient responses

Patients were given a list of symptoms associated with FD and were asked to rate the frequency with which they experience each symptom, using the options of ‘always, often, sometimes, seldom and never’ (Table 1). These responses were then converted to a Likert scale scoring system where always = 4, sometimes = 3, often = 2, seldom = 1 and never = 0, to enable quantification of symptom frequency and severity across the respondent base (Additional file 1: Table S2). Scoring was requested with two different scenarios, symptoms pre-ERT (Table 1) and symptoms post-ERT. The scores for each statement were then compared, which allowed the benefits or burden of treatment to be analysed. Symptoms which had the highest Likert score pre-ERT were ‘burning in the hands and feet’ [31], ‘sensitive to heat and cold’ [30], ‘sweating less than normal’ [30], and ‘pain’ [30], however there were high levels of reporting across the symptoms indicating on overall high and varied symptom burden (Table 1 and Additional file 1: Table S2).

**Table 1:** Self-reported frequency of symptoms pre-ERT, *n* = 11

Symptom Experienced	Pre-ERT				
	Always	Often	Sometimes	Seldom	Never
Burning in the hands and feet	4 (36%)	4 (36%)	1 (9%)	1 (9%)	1 (9%)
Sweating less than normal	5 (45%)	2 (18%)	1 (9%)	2 (18%)	1 (9%)
Sensitive to heat or cold	5 (45%)	2 (18%)	1 (9%)	2 (18%)	1 (9%)
Pain	3 (27%)	4 (36%)	3 (27%)	0 (0%)	1 (9%)
Stomach pain/bloating after eating	2 (18%)	4 (36%)	3 (27%)	1 (9%)	1 (9%)
Tiredness that is not relieved by rest or sleep	2 (18%)	3 (27%)	1 (9%)	4 (36%)	1 (9%)
Feeling sick/being sick	0 (0%)	3 (27%)	4 (36%)	1 (9%)	3 (27%)
Diarrhoea	0 (0%)	1 (9%)	5 (45%)	3 (27%)	2 (18%)
Dizziness	0 (0%)	2 (18%)	2 (18%)	4 (36%)	3 (27%)
Small dark red/purple spots usually found between your belly button and knees	1 (9%)	2 (18%)	0 (0%)	2 (18%)	6 (55%)
Depression/feeling down	0 (0%)	1 (9%)	4 (36%)	0 (0%)	6 (55%)
Ringing in ears	0 (0%)	1 (9%)	3 (27%)	1 (9%)	6 (55%)
Shortness of breath	0 (0%)	0 (0%)	1 (9%)	5 (45%)	5 (45%)
Irregular heartbeat	0 (0%)	0 (0%)	2 (18%)	3 (27%)	6 (55%)
Weight gain/weight loss	0 (0%)	1 (9%)	1 (9%)	3 (27%)	6 (55%)
Cough/wheezing	0 (0%)	0 (0%)	0 (0%)	6 (55%)	5 (45%)
Problems with eyesight	0 (0%)	0 (0%)	0 (0%)	3 (27%)	8 (73%)

Frequency with which patients reported experiencing each listed symptom as occurring before beginning ERT. Patients recorded each symptom as occurring at a frequency of; always, often, sometimes, seldom or never. Both the number of patients and percentage of patients reporting each symptom frequency is shown. Symptoms are ranked in order of most frequently reported pre-ERT

**Fig. 1:**
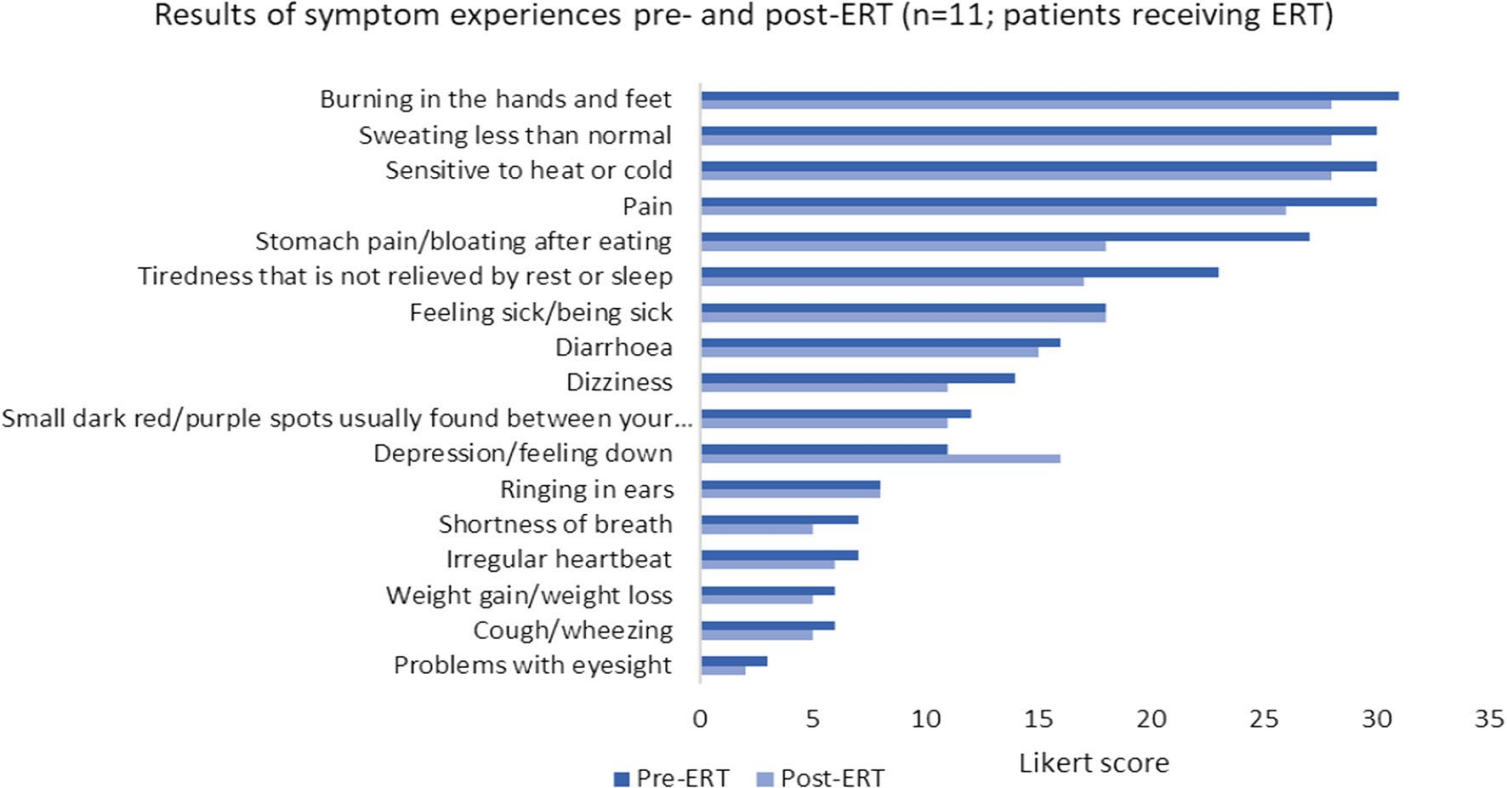
Likert scoring of symptom experience pre-ERT and post-ERT, *n* = 11. Bar chart depicting the frequency with which patients reported experiencing each symptom before beginning ERT (pre-ERT, dark blue) and after starting ERT (post-ERT, light blue). Patients recorded each symptom as occurring at a frequency of; always, often, sometimes, seldom or never. For quantification and comparison this scoring was converted to a Likert scoring, where a score of always = 4, often = 3, sometimes = 2, seldom = 1 and never = 0. Total scores were calculated across the respondents who answered this question, which included those receiving ERT

Overall, treatment caused a 32-point Likert scale improvement in symptomology of the 11 patients who were receiving ERT across all symptoms. Fourteen of the 17 symptoms measured showed an improvement post-ERT when compared to symptom frequency before treatment (Fig. 1 and Additional file 1: Table S2). The symptoms which showed the biggest improvement post-ERT were ‘stomach bloating and pain’ and ‘tiredness not relieved by sleep’. One symptom showed a worsening of symptomology which was ‘depression/feeling down’, however other factors unrelated to FD may influence this symptom. Further, the majority displayed only a limited change i.e., 1 or 2 point improvement.

Each patient was given a series of questions in which they were asked to select the extent to which they agreed or disagreed. Statements were provided with a ten-point rating scale in which 1 = completely disagree and 10 = completely agree with the statement. Of note, only two out of the 14 respondents strongly agreed with the statement ‘I feel good about my health’ by scoring it an 8 or higher, and three out of 14 respondents noted strong disagreement (score of 3 or lower). This aligns with the high symptom burden reported, along with five out of 14 respondents noting that pain can be ‘completely unbearable’ scoring this 10. In general, respondents recorded varied responses relating to the impact of FD on their daily lives, which reflects the different symptom levels experienced by each patient (Additional file 1: Fig. S1).

**Fig. 2:**
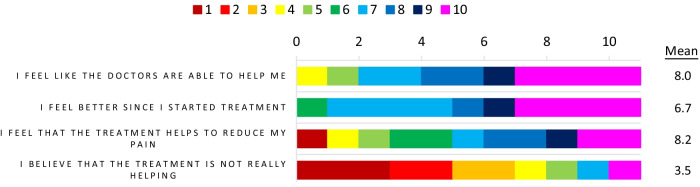
Agreement scores for patients: views towards treatment, n = 11. Patients were asked to rate their agreement with the noted statements based on their views towards treatment, patients who were receiving ERT were the respondent base for these questions. A rating of 1 = completely disagree and 10 = completely agree for all provided statements. The frequency of reporting of each score 1–10 is noted in the bar chart. The bar chart is colour coded as indicated in the key with red/orange indicating low agreement, and blue/pink indicating high agreement for each statement. Note that for the top 3 statements high agreement indicates a positive outcome/outlook for the patient but for the bottom statement it indicated a negative outcome/outlook. The mean values for each statement across the respondents are also shown

Overall, patients had a positive outlook towards their treatment with six of the 11 respondents receiving ERT scoring the statement ‘I feel better since I started treatment’ highly, and five of the 11 patients giving the statement ‘I feel that the treatment helps to reduce my pain’ a high agreement score (Fig. 2). Therefore, while symptom burden was found to be high in the respondent base, there was a feeling that treatment was improving symptomology.

### Caregiver responses

**Fig. 3:**
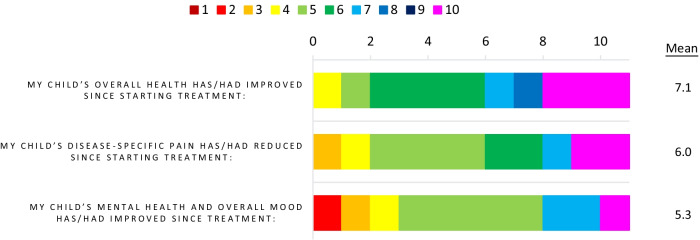
Agreement scores for caregivers: views on ERT, *n* = 11. Caregivers were asked to rate their agreement with the noted statements based on their views towards the effect of ERT, with caregivers whose children were receiving ERT being the respondent base for these questions. A rating of 1 = completely disagree and 10 = completely agree for all provided statements. The frequency of reporting of each score 1–10 is noted in the bar chart. The bar chart is colour coded as indicated in the key with red/orange indicating low agreement, and blue/pink indicating high agreement for each statement. Note that in all cases high agreement would indicate a positive outcome/outlook. The mean values for each statement across the respondents are also shown

To determine the feelings of caregivers towards ERT, questions were posed surrounding their decision-making and, if relevant, the reasons for patients not commencing ERT. In these cases, reasons ranged from ‘did not feel it was the right time to start’, ‘the doctors advised to wait’, and ‘they were offered treatment but declined as was not sure of its benefits’, highlighting a general hesitancy towards initiating the therapy. In five of 11 cases where therapy was being received the caregiver agreed that ‘it was entirely the doctor’s decision for the child to start treatment’. In four out of 11 cases it was noted as a joint decision with family/parent involvement and in two out of 11 cases the caregiver noted that due to their child being male they felt treatment was important, with one specifying ‘it was necessary to start before there are irreversible alterations’. Caregivers were also asked to score statements based on agreement, with an overall positive outlook towards treatment observed (Fig. 3). In particular, high agreement was observed with the statements ‘I am/was aware of the potential side effects of ERT’ and ‘I am/was comfortable that the treatment benefits of ERT outweigh any risks’ (Additional file 1: Fig. S2).

The burden of FD on the caregiver is often represented by the number of days of work missed due to caregiver responsibilities, which could relate to symptom burden or treatment impact. Amongst the respondents, two out of the 14 noted they ‘nearly always’ miss work, with nine out of the 14 respondents noting that they at least ‘sometimes’ miss work (Table 2). Burden was also observed in regard to mental toll, with 10 of the 14 caregivers scoring the statement ‘I often feel guilty and wish I could do more to support my child’ as an 8 or higher on a 1–10 scale.

**Table 2:** Self-reported frequency of missed days of work for caregiver, n = 14

Frequency of missed days of work	How often work missed				
	Nearly Always	Often	Sometimes	Seldom	Never
Total (*n*)	2	1	6	4	1
Total (%)	14.3%	7.1%	42.9%	28.6%	7.1%

Frequency with which caregivers reported missed days of work, both the n and % for each noted frequency is included across the respondent base

**Fig. 4:**
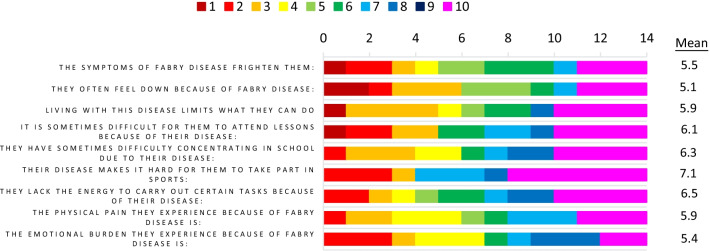
Agreement scores for caregivers: symptom burden from caregiver perspective, *n* = 11. Caregivers were asked to rate their agreement with the noted statements based on the impact of Fabry disease on their child. A rating of 1 = completely disagree and 10 = completely agree for the top 7 provided statements; and a rating of 1 = entirely bearable and 10 = completely unbearable for the bottom 2 provided statements. The frequency of reporting of each score 1–10 is noted in the bar chart. The bar chart is colour coded as indicated in the key with red/orange indicating low agreement, and blue/pink indicating high agreement for each statement. Note that high agreement would indicate a negative outcome/outlook in all cases. The mean values for each statement across the respondents are also shown

High agreement scores were also provided for the following statements; ‘Due to my caregiver responsibilities, I struggle to balance work, family and find time for myself’, ‘The symptoms of FD frighten them (patient)’ and ‘Their disease makes it hard for them to take part in –sports’ (Fig. 4). Overall, responses indicated that the caregivers experience burden themselves but were also conscious of the significant impact to the everyday life of their children. As such, FD impacts the whole family unit, not only through the fact that multiple family members often have the condition, but also due to the increased workload pressures and impact on the caregiver’s ability to work.

### HCP responses

**Fig. 5:**
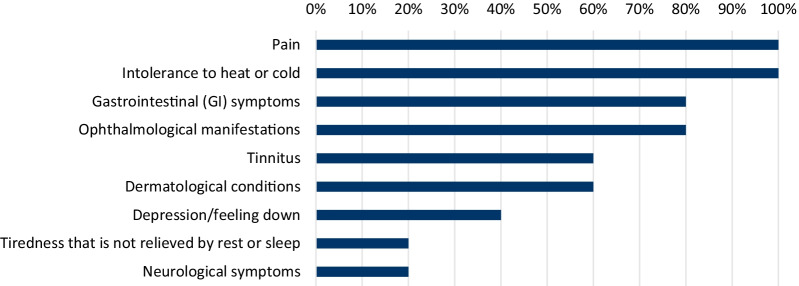
Percentage of physicians reporting as main presenting symptom of Fabry disease in adolescents, *n* = 5. Bar chart indicating the percentage of physicians that selected the noted symptoms as a main presenting symptom of Fabry disease in adolescents. Note multiple responses were allowed

When asked to describe what symptoms were most frequently seen in adolescent patients with FD, all HCPs described ‘intolerance to heat and cold’ and ‘pain’ as the most frequently reported symptoms, in alignment with the responses provided by the patient population. Four of the five HCPs also described GI and ophthalmological manifestations as being frequently reported symptoms (Fig. 5). HCPs reported consistent numbers in regard to the percentage of patients experiencing intolerance to heat or cold (range 40–60%), pain (50–60%) and ophthalmological manifestations (20–30%), with a greater spread in responses given for less frequently reported symptoms. The HCPs also noted the following symptoms as those they expect to see ‘often’ in adolescent patients with FD: intolerance to heat or cold (5/5), pain (4/5), abdominal pain/bloating after eating (4/5), acroparasthesia (4/5) and nausea/vomiting (3/5)**.** When asked ‘How frequently do you see your FD patient’, two HCPs answered ‘More than every 6 months, two answered ‘Every 6 months’ with the final HCP noting a frequency of ‘Every 6 to 12 months’. Overall, results highlight that HCPs feel frequent monitoring of this patient population is required**.**

HCPs were also asked about the current treatment landscape. An average of 43% (range 30–50%) of adolescent patients with FD currently managed at the physicians’ institutions were not receiving treatment. Of those receiving treatment, the most frequent treatment being used was ERT therapy (average of 65%), followed by over-the-counter medication (average of 36%), and other prescribed medicine (overage of 25%). Noted prescribed medications included gabapentin, carbamazepine, and chaperone therapy (clinical trial). HCPs were also asked to describe situations where eligible patients were not prescribed ERT, and the reasoning included; four of the five HCPs stating that the patient did not want to undergo treatment, three of the five HCPs stating that the caregiver did not want the patient to undergo treatment, three of five HCPs stating that the patient could cope without ERT, and two of the five HCPs that the patients could manage symptoms with over-the-counter medications. Responses from the HCPs highlight a reluctance to start ERT as they perceived the potential side effects to outweigh the possible benefits. In line with this, one HCP stated that ERT would ‘likely adversely impact on the patient’s life’.

When asked what factors were prioritised in decision-making regarding whether to prescribe ERT, the ‘severity of patient’s symptoms’ and ‘the impact of treatment on the daily life of the patient’ were most highly ranked with 5/5 and 4/5 HCPs, respectively noting these as their first and second priority (Additional file 1: Table S3). Regarding symptoms prioritised in decision-making, ‘pain’ was the first priority for four of the five HCPs (Additional file 1: Table S4).

When referring to potential challenges in managing adolescent patients with FD, the following were most frequently selected by the respondents; ‘The impact of ERT on QoL of the patient’ (5/5), and ‘The limited efficacy of ERT’ (3/5) (Additional file 1: Table S5). Aligned with the challenges reported and experiences with ERT, in regard to rating statements where 1 = strongly disagree and 10 = strongly agree, the statement with the highest agreement score was ‘ERT imposes high financial burden on the health system’, which had a mean agreement score of 8.4. Further, the second statement with the highest agreement score was ‘There is a high level of unmet need for a treatment with better efficacy’, which had a mean agreement score of 8.2. Additionally, all HCPs selected ‘more manageable treatment options’ as an unmet need.

## Discussion

This study has highlighted the burden experienced by the adolescent population, which we have been able to capture from not only the patient, but also the caregiver and HCP perspectives. The most frequently reported symptoms included ‘pain’, ‘intolerance to heat and cold’ and ‘gastrointestinal symptoms’, which were noted by the patients themselves, as well as HCPs, highlighting consistency between our respondents (Fig. 5). Experiencing pain can have a debilitating impact on everyday life, and often requires concomitant medications for management, which places a burden on the patient and the caregiver. Further, GI symptoms affecting the QoL of the patient can occur such as diarrhoea and poor management of GI symptoms can lead to situations which could be unfavourable for the child. Heat and cold intolerance in adolescents with FD has been linked with an inability to enjoy exercise, sports or ‘play time’ [46], elements that were also seen within our study. Depression is a symptom which also showed an overall lack of improvement in this study, however it is thought depression is a reaction to the symptoms effect on QoL, rather than a symptom of the disease itself [47]. Moreover, the severe symptoms of FD can impact all aspects of daily life, and evidence suggests it can impair social-adaptive functioning, leading to issues in social interactions, school attendance, sports participation, and employment opportunities [48].

The frequently reported symptoms here are in line with previous reporting, confirming the representative nature of the respondent base [49]. The severity of the symptoms was also shown to vary in each patient, which is expected due to the heterogenous nature of the condition. Interestingly, the heterogeneity also extended to the HCP responses in places, which is representative of their particular patient base and past experiences. Importantly, alignment was observed between symptomology and factors contributing to decision-making, with pain noted as a high priority by HCPs, and over a third of respondents reporting that their pain rating at its worst was completely unbearable. The severity of symptoms had a large impact on the QoL impact of the respondents, with the variation observed in responses to the ratings reflective of the symptom heterogeneity in the respondent base. Nonetheless, there were several cases where specific respondents noted high ratings and a severe impact resulting from FD on their QoL, in line with the previously described impact of the highly reported symptoms of this study [49]. Caregivers noted that the adolescent experienced a greater burden than was self-reported, while this could be a misinterpretation from the caregiver it could also reflect under-reporting from the adolescent population and an unwillingness to admit the extent to which their symptoms impact daily life.

Heterogeneity was also observed in reporting of caregiver burden, again highlighting differing symptom severity of the adolescents. However, significant burden was experienced by a subset of the respondents, with impact on their attendance at work and their mental health, reinforcing that FD does not only affect the patient but has wider societal implications. In line with this, direct and indirect financial costs have been shown to be associated with caregiving. Examples of indirect costs attributed to caring for a child with a rare disease includes productivity loss and travel expenses [50]. In this study, many of the caregivers also felt guilty about the QoL they can provide for their child, which may also be influenced by their role in passing on the condition due to its genetic basis. This highlights an unmet need in providing adequate support for the caregiver, as well as the patient, and reinforcing that managing adolescent FD can impact the whole family unit.

Patient and caregiver respondents were positive about ERT, noting an appreciation for the treatment. This aligns with the overall improvement in reported symptomology after receiving ERT, which suggests ERT is efficacious. To date, clinical trials have not, or scarcely, captured the symptom burden from the perspective of the patient and the caregiver in a qualitative nature. The aim of this study was to further add to understanding of self-reported symptoms of FD by the patient, as well as from the perspective of the caregiver and specialist HCPs. A benefit of this study is its ability to allow the patients and caregivers to provide their experiences with existing treatment options, and to provide details of where they saw benefit, and where challenges and unmet needs remained.

The caregivers were comfortable with the benefit to risk balance of ERT and were willing to accept potential side effects or associated burden from the treatment as the disease impact was considered worse. Even the time consumption and absence from school associated with medical care was considered generally acceptable. The HCP respondents provided an alternative perspective, highlighting some of the challenges of ERT. Further, HCPs noted major unmet needs specifically relating to the hope for expanded treatment options. In line with this, management of symptoms also showed variation, with a range of supplemental and concomitant treatments being used in order to reduce the impact of the condition (Additional file 1: Table S1). A possible explanation for the discrepancy between caregivers’ and HCPs’ opinion towards ERT, is HCP awareness of alternative treatments becoming available to the adolescent population and a fuller appreciation of the negatives of treatments. We also note the patient/caregiver and HCP respondents were uncoupled i.e. the HCPs surveyed were not responsible for the care of the patients surveyed, and as such respondents have had or observed different experiences in relation to FD, which will impact their own personal views.

Management of adolescent FD is challenging due to the requirement to balance perceived symptom improvement from treatment, and perceived effect in preventing disease progression, alongside the impact of undertaking treatment. In this study, there was alignment observed between what was reported by caregivers and physicians regarding reasons for not starting ERT, with a hesitancy to initiate treatment noted in certain cases. This was offset by mention of urgency to treat by other respondents, reinforcing the difficult position that caregivers and HCPs are given in deciding on adolescent care. This is also compounded by the understanding of standard of care aiming to prevent progression of the disease, but not being able to impact damage already caused.

Due the rarity of the disease, the presented data is from a small sample size and results should as such be interpreted with caution. There were also other limitations that could have impacted on the data. FD is genetic and a number of caregivers that answered the survey also had FD, as such their own experiences may have impacted the answers given. Use of additional medication by patients may have impacted their reporting of symptom burden post-ERT, however this question was asked in the context of ERT to limit influence. Another limitation, the survey was only performed in three European countries, which limited the data pool. However, given the subjective nature of the survey there is caution from the study team that the cultural and health system differences across regions could also influence response and limit interpretation of data. However, there were no clear distinguishing factors between individual countries that we noted in our data, and due to the small sample size of our study, separating and comparing data from individual countries would not provide a robust insight. Despite the limitations described, consistencies were observed across our dataset and questioning, that is also in alignment with previous reporting [49].

## Conclusions

Overall, our novel study and approach to garner opinion across relevant stakeholders has highlighted there is a significant symptom burden and individual health, development and QoL impact in the adolescent population, which is not fully ameliorated by the current available treatments. The impact of facing a lifelong, incurable and progressive disease has been demonstrated to have physical and psychological consequences and to affect many aspects of daily life [24]. The data from this study demonstrate the unique burden faced by adolescents, which is compounded by the limited treatment guidelines for this patient population [51]. While ERT is an effective treatment and provided symptom relief to many of the respondents in the survey, the respondents still reported significant symptom burden. Further, there was reporting of reluctance to engage in ERT or difficulties associated with the treatment by some. Overall, the findings suggest heterogeneity in symptom presentation requires that the treatment regimen is tailored to the individual, as such physicians need to have a choice of treatment options available.

## Supplementary Information

**Additional file 1: Table S1.** Patient and caregiver characteristics; patients n=14, caregivers n=14. **Table S2. **Accumulated Likert scores for symptoms experienced pre- and post-ERT, n=11. **Table S3. **Factors prioritised when making treatment decisions by HCPs, n=5. **Table S4. **Symptoms prioritised when making treatment decisions by HCPs, n=5. **Table S5. **Top 3 challenges faced by HCPs when treating adolescent patients with FD, n=5. **Fig. S1.** Agreement scores for patients: impact on daily life, n=14. **Fig. S2.** Agreement scores for caregivers: views on ERT side-effects, n=11

## Data Availability

The dataset supporting the conclusions of this article is included within the article. The full dataset from the current study is available from the corresponding author on reasonable request.

## Abbreviations

*α*-Gal A: *α*-Galactosidase A
ERT: Enzyme replacement therapy
FD: Fabry disease
GI: Gastrointestinal
GL-3: Globotriaosylceramide
HCP: Health care professional
IAR: Infusion associated reactions
NBS: Newborn screening
PAO: Patient advocacy organisation
QoL: Quality of life
