# Supplementary material for: Burden associated with Fabry disease and its treatment in 12–15 year olds: results from a European survey
Source: Orphanet J Rare Dis. 2022 Jul 15;17:266. doi: 10.1186/s13023-022-02417-3 (PMC9287883; doi:10.1186/s13023-022-02417-3)
Supplement: Supplementary file 1 — Additional file 1: Table S1. Patient and caregiver characteristics; patients n=14, caregivers n=14. Table S2. Accumulated Likert scores for symptoms experienced pre- and post-ERT, n=11. Table S3. Factors prioritised when making treatment decisions by HCPs, n=5. Table S4. Symptoms prioritised when making treatment decisions by HCPs, n=5. Table S5. Top 3 challenges faced by HCPs when treating adolescent patients with FD, n=5. Fig. S1. Agreement scores for patients: impact on daily life, n=14. Fig. S2. Agreement scores for caregivers: views on ERT side-effects, n=11 [file 13023_2022_2417_MOESM1_ESM.pdf]

Supplemental Table 1. Patient and caregiver characteristics; patients n=14, caregivers n=14

**Patient age at diagnosis**	**Total (%)**
0 – 10 years old	35.7%
11 years old	14.3%
12 years old	0.0%
13 years old	14.3%
14 years old	0.0%
15 years old	14.3%
**Child gender**	
Male	57.1%
Female	42.9%
**Symptoms at diagnosis**	
Yes	42.9%
No	50.0%
Not sure	7.1%
**Child on FD specific treatment**	
Yes	85.7%
No	14.3%

**If on treatment, is it currently ERT**	
Yes	91.7% (78.6% of entire respondent base)
No	8.3% (7.1% of entire respondent base)
**Additional medication taken**	
Yes	100.0%
No	0.0%
Please specify	Ibuprofen, metamizole, tetracaine, amitriptyline, carbamazepine, gabapentin, lamotrigine, multivitamins, vitamin D, melatonin, cetirizine
**Caregiver age group**	
20 - <30 years old	0.0%
30- <40 years old	14.3%
40 - <50 years old	71.4%
Above 50 years old	13.3%
**Caregiver gender**	
Male	21.4%
Female	78.6%

Caregiver relationship to child	
Parent	100.0%
**Caregiver suffers from FD**	
Yes	64.3%
No	35.7%

Responses given to questions by patients or caregivers that provide demographic information on the respondent base. Values are given as a percentage of respondents reporting each answer.

Supplemental Table 2. Accumulated Likert scores for symptoms experienced pre- and post-ERT, n=11

Symptom	Pre-ERT	Post-ERT	Effect of ERT
Burning in the hands and feet	31	28	3 point improvement
Sweating less than normal	30	28	2 point improvement
Sensitive to heat or cold	30	28	2 point improvement
Pain	30	26	4 point improvement
Stomach pain/bloating after eating	27	18	9 point improvement
Tiredness that is not relieved by rest of sleep	23	17	6 point improvement
Feeling sick/being sick	*18*	*18*	*No effect*
Diarrhoea	16	15	1 point improvement

Dizziness	14	11	3 point improvement
Small dark red/purple spots usually found between your belly button and knees	12	11	1 point improvement
Depression/feeling down	*11*	*16*	*5 point worsening*
Ringing in ears	*8*	*8*	*No effect*
Shortness of breath	7	5	2 point improvement
Irregular heartbeat	7	6	1 point improvement
Cough/wheezing	6	5	1 point improvement
Weight gain/weight loss	6	5	1 point improvement
Problems with eyesight	3	2	1 point improvement
**Total**	**279**	**247**	**32 point improvement**

Frequency with which patients reported experiencing each listed symptom before beginning ERT (pre-ERT) and after starting ERT (post-ERT). Patients recorded each symptom as occurring at a frequency of; always, often, sometimes, seldom or never. For quantification and comparison this scoring was converted to a Likert scoring, where a score of always=4, often=3, sometimes=2, seldom=1 and never=0. Total scores were calculated across the respondents who answered this question, which included those receiving ERT. Comparing the total score pre-ERT and post-ERT demonstrates the overall change, most symptoms showed an improved rating, those that did not are in italics. Symptoms are ranked in order of most frequently reported pre-ERT.

Supplemental Table 3. Factors prioritised when making treatment decisions by HCPs, n=5

Rank	1st	2nd	3rd	4th	5th	Score (1st = 5 points, 5th = 1 point)
Severity of the patient's symptoms	80%	20%	0%	0%	0%	24
Impact of treatment on daily life of the patient	20%	60%	20%	0%	0%	20
Likely adherence to the treatment regimen	0%	20%	0%	60%	20%	11
Willingness of the caregiver to initiate treatment	0%	0%	40%	40%	20%	11
Age of the patient	0%	0%	40%	0%	40%	8
Not answered	0%	0%	0%	0%	20%	1

Percentage of physicians that selected each indicated option as a factor that they prioritise when making treatment decisions for adolescent patients with Fabry disease. Physicians ranked these factors as their 1st to 5th priority as indicated. To enable ranking of the most prioritised factor noted by physicians across the respondent base, their ranking was converted to a numerical score with a ranking of 1st=5 points, 2nd=4 points, 3rd=3 points, 4th=2 points and 5th=1 point. Options are ordered with the most highly prioritised factor at the top.

Supplemental Table 4. Symptoms prioritised when making treatment decisions by HCPs, n=5

Rank	1st	2nd	3rd	4th	5th	Score (1st = 5 points, 5th = 1 point)
Pain	80%	20%	0%	0%	0%	24
GI symptoms	0%	60%	20%	0%	0%	15
Intolerance to heat or cold	0%	20%	60%	20%	0%	15

Tiredness that is not relieved by rest or sleep	0%	0%	20%	60%	0%	9
Neurological symptoms	20%	0%	0%	0%	40%	7
Ophthalmological manifestations	0%	0%	0%	20%	0%	2
Renal problems	0%	0%	0%	0%	40%	2
Depression/feeling down	0%	0%	0%	0%	20%	1

Percentage of physicians that selected each indicated option as a symptom that they prioritise when making treatment decisions for adolescent patients with Fabry disease. Physicians ranked these symptoms as their 1st to 5th priority as indicated. To enable ranking of the most prioritised symptom noted by physicians across the respondent base, their ranking was converted to a numerical score with a ranking of 1st=5 points, 2nd=4 points, 3rd=3 points, 4th=2 points and 5th=1 point. Options are ordered with the most highly prioritised symptom at the top.

Supplemental Table 5. Top 3 challenges faced by HCPs when treating adolescent patients with FD, n=5

**Rank**	**1st**	**2nd**	**3rd**	**Score (1st = 3 points, 3rd = 1 point)**
Impact of ERT on the quality of life of the patient	40%	20%	40%	10
Limited ERT efficacy	40%	20%	0%	7
Difficulty of diagnosis	20%	0%	20%	4
Lack of available treatment options	0%	20%	20%	3
Lack of individualised treatment guidelines	0%	20%	0%	2
Other *(please specify)*	0%	20%	0%	2
Patients not adhering to the treatment regimen	0%	0%	20%	1

Percentage of physicians that selected each indicated option as a challenge in treating adolescent patients with Fabry disease. Physicians ranked these challenges as their 1st, 2nd or 3rd greatest challenge as indicated. To enable ranking of the greatest challenges noted by

physicians across the respondent base, their ranking was converted to a numerical score with a ranking of 1st=3 points, 2nd=2 points and 3rd=1 point. Options are ordered with the most highly ranked challenge at the top.

Supplemental Figure 1. Agreement scores for patients: impact on daily life, n=14

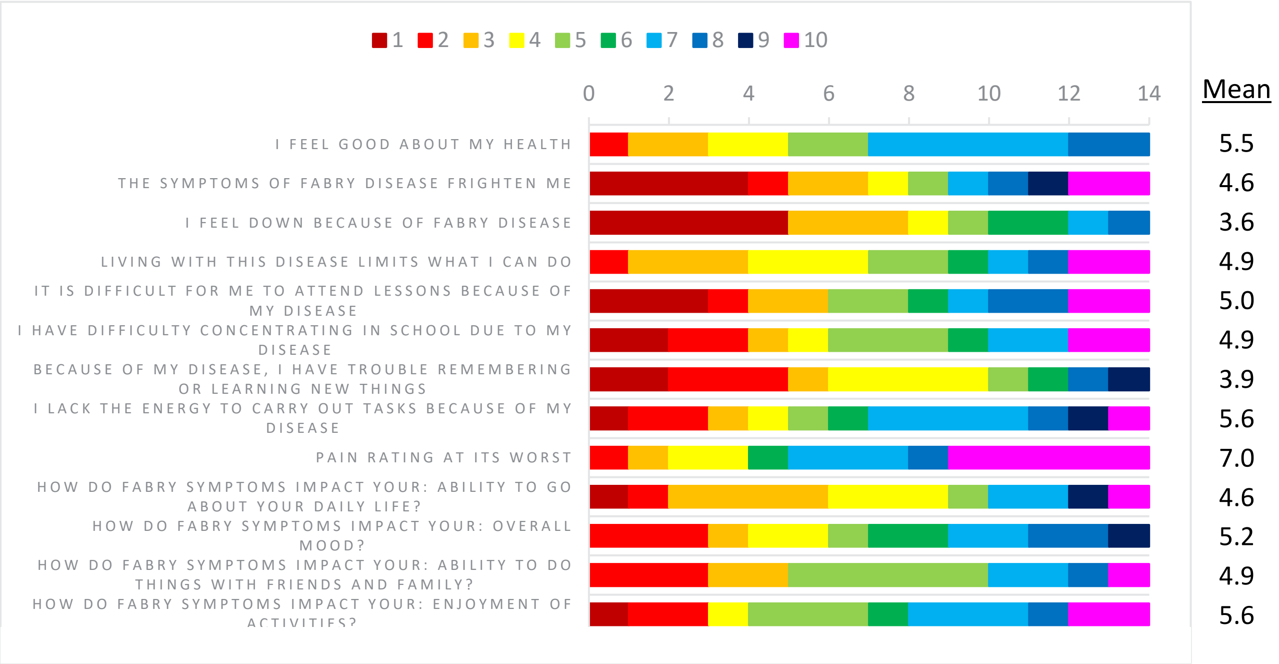

Patients were asked to rate their agreement with the noted statements based on the impact of Fabry disease on their daily life.

A rating of 1=completely disagree and 10=completely agree for the top 8 provided statements; a rating of 1=entirely bearable and 10=completely bearable for the pain rating; and a rating of 1=no impact and 10=completely interferes for the bottom 4 provided statements. The frequency of reporting of each score 1-10 is noted in the bar chart. The bar chart is colour coded as indicated in the key with red/orange indicating low agreement, and blue/pink indicating high agreement for each statement. Note that for the top statement, high agreement would indicate a positive outcome/outlook for the patient and in all other cases would indicate a negative outcome/outlook. The mean values for each statement across the respondents are also shown.

Supplemental Figure 2. Agreement scores for caregivers: views on ERT side-effects, n=11

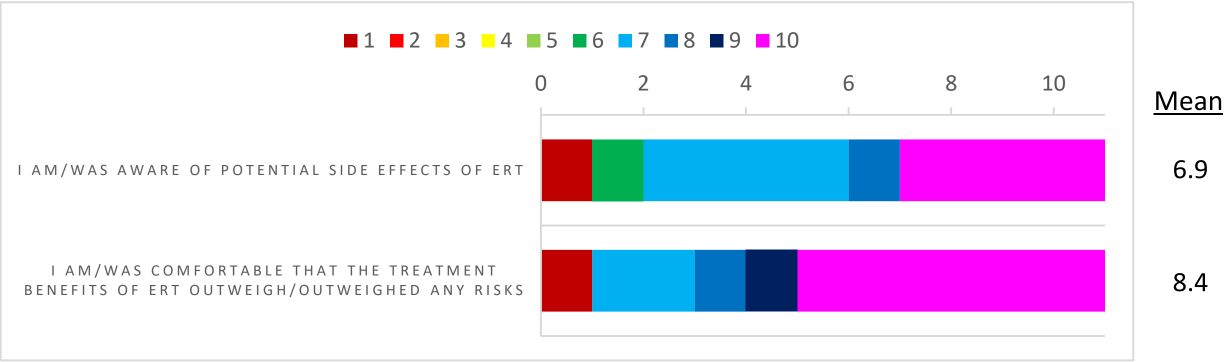

Caregivers were asked to rate their agreement with the noted statements based on their views relating to ERT side-effects, respondents who reported that their child was receiving ERT therapy were the respondent base for this question. A rating of 1=completely disagree and 10=completely agree for the provided statements. The frequency of reporting of each score 1-10 is noted in the bar chart. The bar chart is colour coded as indicated in the key with red/orange indicating low agreement, and blue/pink indicating high agreement for each statement. Note that high agreement would indicate a positive outcome/outlook. The mean values for each statement across the respondents are also shown.
